# Correlation between compliance and brace treatment in juvenile and adolescent idiopathic scoliosis: SOSORT 2014 award winner

**DOI:** 10.1186/1748-7161-9-6

**Published:** 2014-06-13

**Authors:** Angelo G Aulisa, Marco Giordano, Francesco Falciglia, Emanuele Marzetti, Andrea Poscia, Vincenzo Guzzanti

**Affiliations:** 1 U.O.C. of Orthopedics and Traumatology, Children’s Hospital Bambino Gesù, Institute of Scientific Research, P.zza S. Onofrio, Rome 4-00165, Italy; 2 Department of Orthopedics, University Hospital “Agostino Gemelli”, Catholic University of the Sacred Heart School of Medicine, Rome 00168, Italy; 3 Institute of public health, University Hospital “Agostino Gemelli”, Catholic University of the Sacred Heart School of Medicine, Rome 00168, Italy; 4 University of Cassino, Cassino, FR 03043, Italy

**Keywords:** Compliance, Juvenile idiopathic scoliosis, Adolescent idiopathic scoliosis, PASB brace, Conservative treatment

## Abstract

**Background:**

Over the last years, evidence has accumulated in support of bracing as an effective treatment option in patients with idiopathic scoliosis. Yet, little information is available on the impact of compliance on the outcome of conservative treatment in scoliotic subjects. The aim of the present study was to prospectively evaluate the association between compliance to brace treatment and the progression of scoliotic curve in patients with idiopathic adolescent (AIS) or juvenile scoliosis (JIS).

**Methods:**

Among 1.424 patients treated for idiopathic scoliosis, 645 were eligible for inclusion criteria. Three outcomes were distinguished in agreement with the SRS criteria: curve correction, curve stabilization and curve progression. Brace wearing was assessed by one orthopaedic surgeon (LA) and scored on a standardized form. Compliance to treatment was categorized as complete (brace worn as prescribed), incomplete A (brace removed for 1 month), incomplete B (brace removed for 2 months), incomplete C (brace removed during school hours), and incomplete D (brace worn overnight only). Chi square test, T test or ANOVA and ANOVA for repeated measures tests were used as statistical tests.

**Results:**

The results from our study showed that at follow-up the compliance was: Complete 61.1%; Incomplete A 5.2%; Incomplete B 10.7%; Incomplete C 14.2%; Incomplete D 8.8%. Curve correction was accomplished in 301/319 of Complete, 19/27 Incomplete A, 25/56 Incomplete B, 52/74 Incomplete C, 27/46 Incomplete D. Cobb mean value was 29.8 ± 7.5 SD at beginning and 17.1 ± 10.9 SD at follow-up. Both Cobb and Perdriolle degree amelioration was significantly higher in patients with complete compliance over all other groups, both in juvenile, both in adolescent scoliosis. In the intention-to-treat analysis, the rate of surgical treatment was 2.1% among patients with definite outcome and 12.1% among those with drop-out. Treatment compliance showed significant interactions with time.

**Conclusion:**

Curve progression and referral to surgery are lower in patients with high brace compliance. Bracing discontinuation up to 1 month does not impact on the treatment outcome. Conversely, wearing the brace only overnight is associated with a high rate of curve progression.

## Background

Idiopathic scoliosis is a 3-dimensional spine deformity affecting 0.3–0.5% of children younger than 16 years of age [1,2]. Bracing is frequently prescribed as a non-surgical treatment option to patients with idiopathic scoliosis and a spinal curvature greater than 20° Cobb. Notably, bracing is the only conservative approach with proven effectiveness in such condition [3-8]. Several factors may have influence the efficacy of brace treatment, including age, gender, bone maturity, prescribed hours of bracing, and curve pattern and magnitude. In particular, a recent Cochrane Review [9-11] and a meta-analysis by Rowe [12] have shown that compliance has a great impact on the outcome of bracing. Indeed, the guidelines released by the Society on Scoliosis Orthopedic and Rehabilitation Treatment (SOSORT) indicate adherence as a key element in determining the efficacy of bracing [13,14].

Given these considerations, the evaluation of compliance to bracing through the use of either dedicated questionnaires [15-18] or the application of sensors within the brace [13,19-25], increases the internal validity of clinical investigations on the topic. The aim of the present study was to prospectively evaluate the association between compliance to bracing and the progression of scoliotic curve, including surgery referral rate, in patients with idiopathic adolescent and juvenile scoliosis.

## Methods

### Study design and participants

#### Patients

This is a prospective study based on ongoing database including 1,424 patients treated for idiopathic scoliosis. Of these 522 patients whit a definite outcome were included, while 120 were excluded due to premature bracing discontinuation. Inclusion criteria were as follows: Risser 0–2 at the beginning of treatment, curve magnitude (C_M_) 20°-50° Cobb, full-time brace prescription and no previous treatment. Curves between 20° and 25° Cobb degrees were included only in the presence of documented progression. The latter was assessed on two consecutive X-rays taken at 6-month interval and was defined as an increase greater than 5° in C_M_ (Cobb's method) [26]. The minimum duration of follow-up was 24 months after the end of treatment. Compliance to treatment was categorized as: brace worn as prescribed (complete), brace removed for 1 month of a year (incomplete A), brace removed for 2 months of a year (incomplete B), brace removed during school hours (incomplete C), and brace worn overnight only (incomplete D).

### Bracing protocol

All patients were prescribed with full-time bracing (i.e., max 22 hours daily, min 18 hours daily). The patients with thoracolumbar and lumbar curves were treated with PASB [27], instead thoracic and double major curves with Lyon or Milwaukee Brace in according to age. In order to maximize treatment adherence, patients were always followed by the same physician. Controls were performed every two months until Risser 3, and every three months, always in the presence of parents. Close checks were also necessary to maximize the efficacy of bracing over time. Frequent checks allowed verifying and implementing compliance by establishing an open and friendly relationship with the patients and their parents.

Weaning was started when ring-apophysis fusion was seen to begin on a latero-lateral (LL) radiograph view [28], which corresponds to a Risser sign 4 or 5 on an antero-posterior (AP) standing radiograph view. Weaning consisted of 2 to 4 hours bracing reduction at 2-month intervals.

Compliance to treatment was established via in-person interviews at each clinical examination. Responses of patients were ratified by their parents and indirectly by the assessment of the hump course [29]. In cases of hump or curve worsening, a thorough evaluation of the patient's behaviour was performed and a stronger parental involvement encouraged.

### Endpoints

For the present study, only the X-ray performed at conventional times were considered: beginning of treatment (t_1_), 4–6 months after the beginning of treatment (t_2_), intermediate time between t_1_ and t_4_ (t_3_), end of weaning (t_4_), 2-year minimum follow-up (t_5_). For each patient, AP and LL view standing X-rays of the whole spine were performed. X-rays before treatment (t_1_) as well as those at t_4_ and t_5_ were taken while out of brace. All other radiographic controls were performed with the patient wearing the brace, in order to assess its corrective action. The first X-ray was obtained at 4–6 months from the beginning of treatment. All other controls were performed once a year. The AP view was used to determine the patient's skeletal age (Risser's sign) and to obtain the C_M_ and torsion of the apical vertebra (T_A_: Perdriolle's method). Measurements were obtained by two independent observers. The end-vertebrae were pre-selected to reduce inter-observer error [26]. Curves were classified according to SRS in thoracic, thoracolumbar, lumbar, and double major. As recommended by the SRS Committee on Bracing and Non-operative Management, outcomes were classified as follows: (1) correction (percentage of patients with ≤ 5° curve progression), (2) stabilization (percentage of patients with > −5 and < 5°changes in C_M_), (3) progression (percentage of patients with ≥ 5° progression at maturity), and (4) percentage of patients with curves exceeding 45° at maturity and those who were recommended for or had undergone surgery.

### Statistical analysis

Descriptive analyses, independent t-tests and chi-square tests were used to describe and analyze the characteristics of the study population at baseline, as appropriate.

Differences between baseline (t1) and the end of the study (t5) in Cobb and Perdriolle degrees over compliance with treatment was tested using One Way ANOVA adjusted for Bonferroni.

The influence of possible confounders on Cobb and Perdriolle degree was tested with a multiple linear regression model. The model was run separately for juvenile and adolescent scoliosis using the differences between baseline (t1) and the end of the study (t5) in Cobb and Perdriolle degrees as dependent variable and orthopaedic corset compliance as independent variable. The model was adjusted for: gender, age at baseline, length of treatment, type of brace and, respectively, Cobb and Perdriolle degree at baseline.

Furthermore, ANOVA for repeated measures with the last observation carried forward (LOCF) was used to examine the within and between group effects on the dependent variable.

The Anova was repeated separately for juvenile and adolescent scoliosis using the Cobb and Perdriolle degree, respectively, as dependent variable. Time from baseline (t1) to t5 and the orthopedic corset compliance was used to define the within and between groups variability. Assumptions of repeated measures were tested; when sphericity assumption was violated, Greenhouse-Geisser adjustments were applied.

Statistical significance was set as a p value ≤0.05 (two-tailed). The analyses were performed by using SPSS 13.0 software.

## Results

Sample characteristics according the type of scoliosis are displayed in Table 1. The age at baseline [mean (SD)] was 9.0 (1.2) and 12.6 for Juvenile and adolescent scoliosis, while the length of treatment was 80.3 (26.2) month and 57.2 (18.4), respectively. The results from our study showed that at follow-up the compliance was: brace worn as prescribed (Complete) 61.1%; brace removed for 1 month of a year (incomplete A) 5.2%; brace removed for 2 months of a year (incomplete B) 10.7%; brace removed during school hours (incomplete C) 14.2%; brace worn overnight only (incomplete D) 8.8%. Curve correction was accomplished in 301/319 of Complete, 19/27 brace removed for 1 month of a year (incomplete A), 25/56 brace removed for 2 months of a year (incomplete B), 52/74 brace removed during school hours (incomplete C), 27/46 brace worn overnight only (incomplete D). Surgery Referral was 3/319 of Complete, 1/27 brace removed for 1 month of a year (incomplete A), 4/56 brace removed for 2 months of a year (incomplete B), 2/74 brace removed during school hours (incomplete C), 4/46 brace worn overnight only (incomplete D).

**Table 1:** Sample characteristics

		**JIS**	**AIS**
Gender	Male	116	367
	Female	11	28
Curve	Double major	5	11
	Lumbar	24	96
	Thoracic	34	77
	Thoracolumbar	64	211
Brace	Lion	18	138
	Milw	52	42
	Pasb	57	215
Compliance	Complete	66	253
	Incomplete A	12	15
	Incomplete B	25	31
	Incomplete C	14	60
	Incomplete D	10	36
Total (522)		127	395

Cobb mean value was 29.8 ± 7.5 SD at beginning and 17.1 ± 10.9 SD at follow-up. Perdriolle was 13.2 ± 5.6 SD at beginning and 7.6 ± 4.8 at follow-up. Differences in Cobb and Perdriolle degrees between baseline (t1) and end of study (t5) over compliance with brace treatment are showed in Table 2 and 3, respectively.

**Table 2:** COBB degrees distribution according the treatment adherence

**Juvenile scoliosis (JIS)**					
**Compliance (N)**		**Baseline**	**t5**	**Difference between t5 and baseline**	**Difference over compliance**
**Complete**	mean	29.4	10.9	−18.5	Reference
	SD	6.7	9.5	(p < 0.001)	
**Incomplete A**	mean	29.2	21.8	−7.4	11.1
	SD	6.9	9.1	(P = 0.006)	(p < 0.001)
**Incomplete B**	mean	29.2	24.9	−4.1	14.4
	SD	6.8	10.9	(p = 0.045)	(p < 0.001)
**Incomplete C**	mean	28.8	18.4	−10.4	8.1
	SD	8.1	5.5	(p < 0.001)	(p < 0.001)
**Incomplete D**	mean	32.6	24.2	−8.4	10.1
	SD	7.5	6.2	(p < 0.001)	(p < 0.001)
**Total**	mean	29.5	16.5	−13	
	SD	6.9	11.0	(p < 0.001)	
**Adolescent scoliosis (AIS)**					
**Compliance (N)**		**Baseline**	**t5**	**Difference between t5 and baseline**	**Difference over compliance**
**Complete**	mean	30.5	13.1	−17.4	Reference
	SD	8.0	10.0	(p < 0.001)	
**Incomplete A**	mean	30.5	20.8	−9.7	7.7
	SD	5.1	6.8	(p < 0.001)	(p < 0.001)
**Incomplete B**	mean	28.6	24.2	−4.4	13
	SD	7.0	8.0	(p = ns)	(p < 0.001)
**Incomplete C**	mean	30.2	20.1	−10.1	7.3
	SD	6.7	7.5	(p < 0.001)	(p < 0.001)
**Incomplete D**	mean	31.5	23.1	−8.4	9
	SD	8.8	9.0	(p < 0.001)	(p < 0.001)
**Total**	mean	30.4	16.3	−14.1	
	SD	7.7	10.2	(p < 0.001)	

**Table 3:** Perdriolle degrees distribution according the treatment adherence

**Juvenile scoliosis (JIS)**					
**Compliance (N)**		**Baseline**	**t5**	**Difference between t5 and baseline**	**Difference over compliance**
**Complete**	mean	12.2	6.0	−6.2	Reference
	SD	4.1	5.6	(p < 0.001)	
**Incomplete A**	mean	13.0	10.3	−2.7	3.5
	SD	5.0	5.7	(p = 0.049)	(p = 0.013)
**Incomplete B**	mean	14.1	12.5	−1.6	4.6
	SD	4.7	6.1	(p = 0.199)	(p < 0.001)
**Incomplete C**	mean	10.2	8.5	−1.7	4.5
	SD	2.8	2.3	(p = 0.143)	(p = 0.001)
**Incomplete D**	mean	14.6	14.3	−0.3	5.9
	SD	5.4	5.2	(p = 0.766)	(p < 0.001)
**Total**	mean	12.9	8.7	−4.2	
	SD	4.9	6.2	(p < 0.001)	
**Adolescent scoliosis (AIS)**					
**Compliance (N)**		**Baseline**	**t5**	**Difference between t5 and baseline**	**Difference over compliance**
**Complete**	mean	12.0	6.2	−5.8	Reference
	SD	4.1	5.2	(p < 0.001)	
**Incomplete A**	mean	14.9	11.2	−3.7	2.1
	SD	4.7	6.6	(p < 0.001)	(p < 0.001)
**Incomplete B**	mean	12.9	11.4	−1.5	4.3
	SD	4.1	5.6	(p < 0.09)	(p < 0.001)
**Incomplete C**	mean	14.4	10.9	−3.5	2.3
	SD	4.9	5.9	(p < 0.001)	(p < 0.001)
**Incomplete D**	mean	13.7	10.9	−2.8	3.0
	SD	5.9	20.0	(p < 0.001)	(p < 0.001)
**Total**	mean	12.8	8.3	−4.5	
	SD	4.6	8.3	(p < 0.001)	

**Figure 1:**
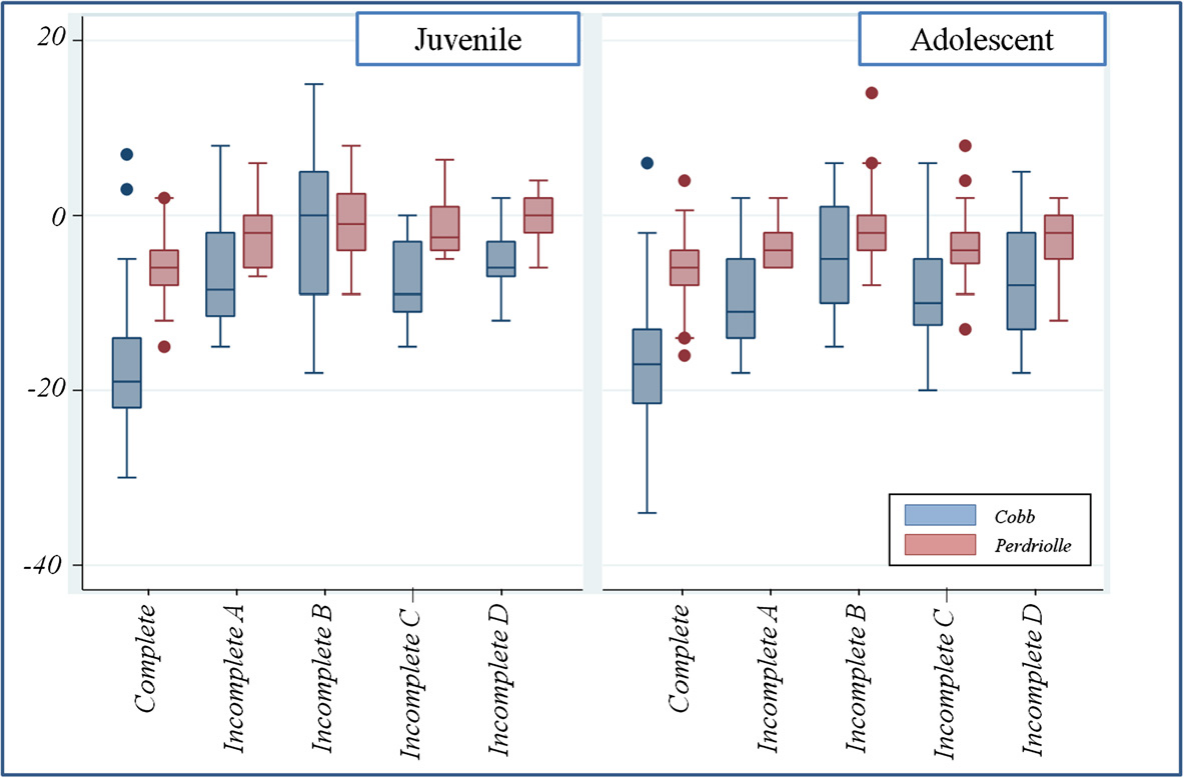
Difference between t5 and t1 in Cobb degree (blue boxes) and Perdriolle degree (red boxes) over type of scoliosis and Compliance (0 = complete; 1 = brace removed for 1 month of a year (incomplete A); 2 = brace removed for 2 months of a year (incomplete B); 3 = brace removed during school hours (incomplete C); 4 = Incomplete D).

The Figure 1 showed the difference between t5 and t1 in Cobb and Perdriolle degrees over type of scoliosis and Compliance. Both Cobb and Perdriolle degree amelioration was significantly higher in patients with complete compliance over all other groups, both in juvenile, both in adolescent scoliosis. In adolescent scoliosis, also patients that removed brace for 2 months of a year (incomplete B) showed higher mean differences than those that removed brace during school hours (incomplete C).

Univariate analysis at baseline have shown significant differences over adherence groups only between type of brace and Perdriolle degree in patient with adolescent scoliosis (higher use of PASB (p = 0.02) and lower Perdriolle degree (p = 0.0002) in patient with complete compliance). No significant differences were found according to gender and type of curve. Similarly, no differences were found in Cobb degree at baseline over compliance groups.

Multiple regression [Tables 4 and 5] showed that compliance with treatment was significantly associated with greater improvement in Cobb and Perdriolle degrees. Length of treatment was always significantly associated with a lower reduction, while age and Cobb degree at baseline were associated respectively with a lower and higher Cobb degrees reduction only in adolescent scoliosis. PASB brace was significantly associated with a higher Cobb and Perdriolle reduction in adolescent scoliosis.

**Table 4:** Multiple linear regression: results for Cobb degree difference (t5 vs baseline)

**Cobb degree difference**		**JIS (R2 = 0.55)**			**AIS (R2 = 0.48)**		
**Independent variables**		**B coefficient**	**Standard error**	**p**	**B coefficient**	**Standard error**	**p**
Adherence	Complete (reference)	-	-	-	-	-	-
	Incomplete A	8.57	2.36	0.000	6.54	1.49	0.000
	Incomplete B	13.77	1.78	0.000	11.39	1.08	0.000
	Incomplete C	7.30	2.43	0.003	6.88	0.83	0.000
	Incomplete D	10.35	2.53	0.000	8.60	1.02	0.000
Gender		4.02	2.53	0.114	0.85	1.11	0.444
Age baseline		0.99	0.61	0.109	0.87	0.25	0.001
Length of treatment		0.08	0.03	0.008	0.11	0.02	0.000
Cobb at baseline		−0.11	0.11	0.321	−0.14	0.04	0.001
Type of brace	Lyon (Reference)	-	-	-	-	-	-
	Milwaukee	0.76	2.14	0.724	−0.29	1.01	0.776
	Pasb	−1.76	2.21	0.428	−2.61	0.65	0.000

**Table 5:** Multiple linear regression: results for Perdriolle degree difference (t5 vs baseline)

**Perdriolle degree difference**	**JIS (R2 = 0.40)**			**AIS (R2 = 0.29)**		
**Independent variables**	**B coefficient**	**Standard error**	**p**	**B coefficient**	**Standard error**	**p**
	Complete (reference)	-	-	-	-	-
Adherence	Incomplete A	1.92	1.19	0.109	1.35	0.78
	Incomplete B	4.23	0.89	0.000	3.97	0.56
	Incomplete C	3.35	1.22	0.007	1.94	0.44
	Incomplete D	4.65	1.27	0.000	2.46	0.54
Gender		2.15	1.29	0.099	0.18	0.58
Age baseline		0.18	0.31	0.558	0.09	0.13
Length of treatment		0.05	0.01	0.001	0.05	0.01
Perdriolle at baseline		−0.04	0.08	0.633	-0.02	0.04
Type of brace	Lyon (Reference)	-	-	-	-	-
	Milwaukee	−0.18	1.08	0.865	0.21	0.52
	Pasb	−0.47	1.09	0.669	−0.93	0.34

### Juvenile

**Figure 2:**
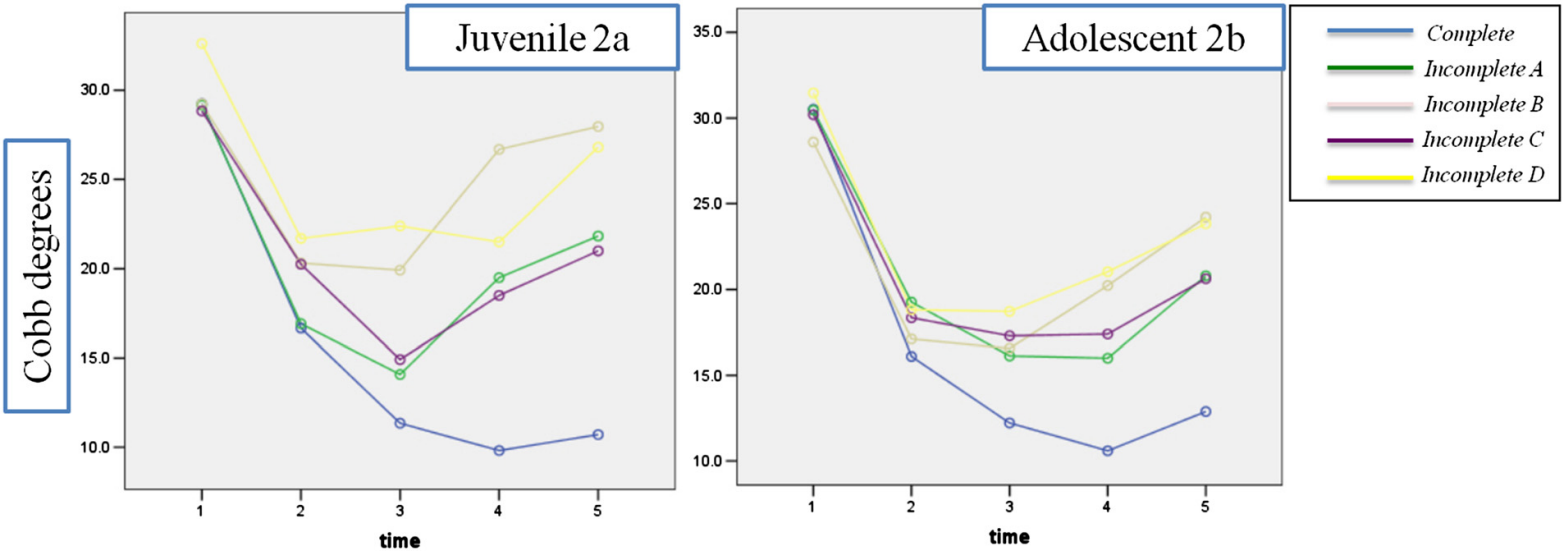
Changes in Cobb degrees for each level of compliance during each time point.

The evaluation of Cobb degrees for each level of compliance during each time point in the juvenile curves showed the following results [Figure 2a]. The assumption of sphericity was not met, so all comparisons were made using the Greenhouse–Geisser correction. There was a significant main effect for time (p = 0.000), for compliance (p = 0.003) and for the Interaction (p = 0.000). All time showed within-group differences in the COBB degree, except time 2 versus time 3. All groups showed differences in Cobb degree, but they were significative only between compliance Complete and Incomplete B (brace removed for 2 months of a year ) (−9.268; P = 0.000) and Complete and Incomplete D (brace worn overnight only) (−9.444; p = 0.013). Significant interactions showed that the effect of time was different for the five compliance groups: as it is reasonable, the maximum compliance had greater Cobb degree reduction than the other groups during time, especially in late treatment. There was significant greater improvement in COBB degree favouring "complete compliance" group respect those that worn brace overnight only (incomplete D) at many time points: t3, t4 and t5 (P < 0.01). Patients with brace removed for 1 month of a year (incomplete A), showed a greater improvement than those that worn brace overnight only (incomplete D) at t3, without reach the statistical significance (P = 0.052).

**Figure 3:**
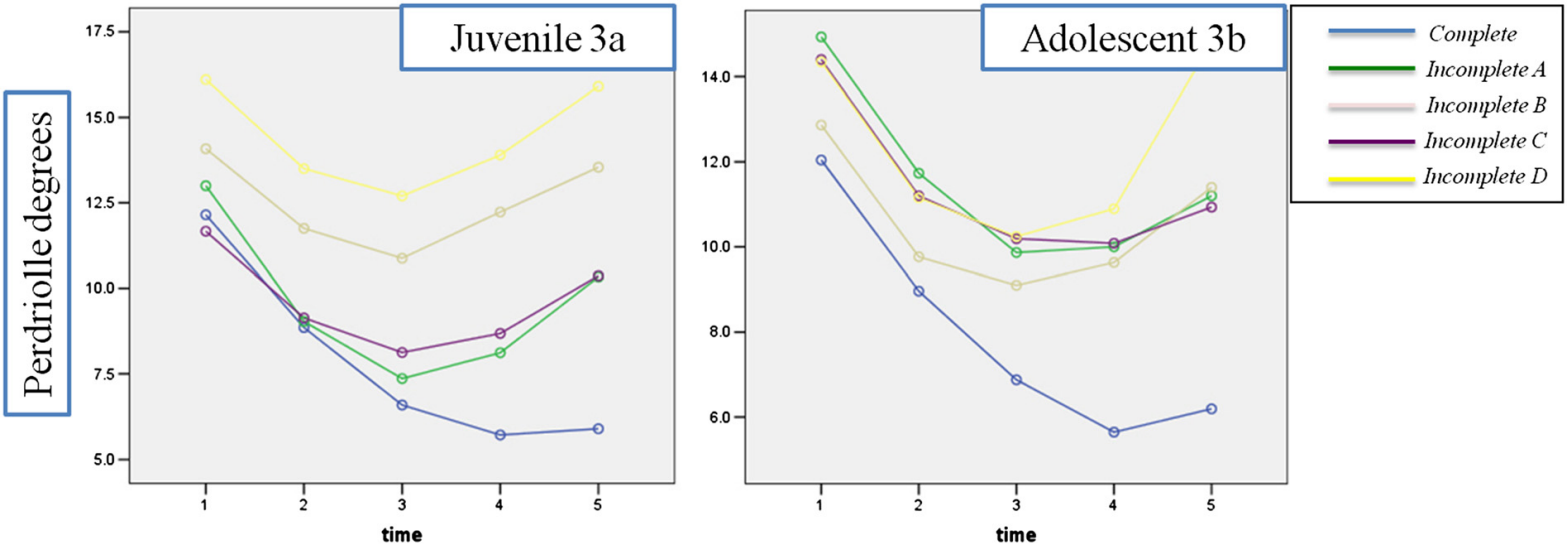
Changes in Perdriolle degrees for each level of compliance during each time point.

While the Perdriolle showed these results [Figure 3a]. The assumption of sphericity was not met, so all comparisons were made using the Greenhouse–Geisser correction. There was a significant main effect for time, compliance and Interaction (p = 0.000). There was within-group differences in the COBB degree for all time, except t2 versus t4 and t5 and t3 versus t4. All groups showed a reduction in Cobb degree, but there was a significant difference only between compliance Complete and Incomplete B (brace removed for 2 months of a year) (−4.658; P = 0.004) and Incomplete D (brace worn overnight only) (−6.582; P = 0.005). Significant interactions showed that the effect of time was different for the compliance groups: as it is shown in Figure 3, the higher interaction was between compliance complete and incomplete C (brace removed during school hours). There was significant greater improvement in COBB degree favouring different compliance group versus Compliance D at many time points: Complete compliance in all time [t1 (P = 0,018); t2 (P = 0.009); t3 (P = 0.002), t4 (P = 0.000), t5 (P = 0.000)]; Incomplete A in t2, t3, t4 and t5; Incomplete C in t1 (P = 0.033), t2 (P = 0.049), t4 (P = 0.022) and t5 (P = 0.047).

### Adolescent

The evaluation of Cobb degrees for each level of compliance during each time point in the adolescent curves showed the following results [Figure 2b]. The assumption of sphericity was not met, so all comparisons were made using the Greenhouse–Geisser correction. There was a significant main effect for time, for compliance and for the Interaction (p = 0.000). There was within-group differences in the COBB degree for all time, except t2 versus t4 and t3 versus t4. All groups showed a reduction in Cobb degree, but the group with complete compliance had significant differences with all the other except of subgroup that removed brace for 1 month of a year (incomplete A), (−4.070; P = 0.481). Significant interactions showed that the effect of time was different for the five compliance groups: as it is reasonable, the maximum compliance had greater Cobb degree reduction than the other groups during time, especially in late treatment. There was significant greater improvement in COBB degree favouring "complete compliance" group respect Incomplete D (brace worn overnight only) at many time points: t3, t4 and t5 (P < 0.01). Furthermore the group that removed brace for 1 month of a year (incomplete A) showed a greater improvement than those that worn brace overnight only (incomplete D) at t2 and t4, without reach the statistical significance (P = 0.058 and 0.055 respectively); subgroup that removed brace during school hours (incomplete C) showed a greater improvement at t4 (P = 0.043).

While the Perdriolle showed these results [Figure 3b]. The assumption of sphericity was not met, so all comparisons were made using the Greenhouse–Geisser correction. There was a significant main effect for time, compliance and Interaction (p = 0.000). There was within-group differences in the Cobb degree for all time, except t2 vs t5. All groups showed a reduction in Perdriolle degree, but the group with complete compliance had significant differences with all the other except that "brace removed for 1 month of a year (incomplete A)" compliance (−3.090; P = 0.10). Significant interactions showed that the effect of time was different for the five compliance groups: as it is reasonable, the maximum compliance had greater Perdriolle degree reduction than the other groups during time, especially in late treatment. There was significant greater improvement in COBB degree favouring "complete compliance" group respect worn brace overnight only (incomplete D) at many time points: t1 (P = 0.035) t3 (P = 0.002), t4 (P = 0.000) and t5 (P = 0.001)

### Intention to treat analysis

A total of 642 patients were eligible for inclusion criteria. Of these, 522 patients have a definite outcome and 14 of these have indication to surgery, while 120 have abandon the treatment. 65 of 120 patients who abandoned the treatment were revaluate at follow up and only 9 of these have indication to surgery (Table 6).

**Table 6:** Sample of patients that abandon the treatment

	**Abandon without F-up no./total no. (%)**	**Abandon with F-up no./total no. (%)**
Risser 0	1/55 (1,9%)	2/65 (1,5%)
Risser 1	3/55 (5,4%)	1/65 (3%)
Risser 2	12/55 (21,8%)	8/65 (12,5%)
Risser 3	37/55 (67,2%)	40/65 (61,5%)
Risser 4	2/55 (3,6%)	14/65 (21,5%)
Curve correction at time of abandon	38/55(69,1%)	55/65 (84,6%)
Curve stabilization at time of abandon	15/55 (27,3%)	4/65 (6,1%)
Curve progression at time of abandon	2/55 (3,6%)	6/65 (9,3%)
Curve correction at follow-up	-	19/65 (29,2%)
Curve stabilization at follow-up	-	30/65 (46,2%)
Curve progression at follow-up	-	16/65 (24,6%)
Indication to surgery	55/55 (100%)	9/65 (13,8%)

The rate of surgery referral was 2.1% among patients with definite outcome and 12.1% among those with drop-out.

## Discussion

The aim of the present study was to evaluate the relationship between compliance to bracing wear and the progression of scoliotic curves in patients with idiopathic adolescent (AIS) or juvenile scoliosis (JIS) treated with PASB, Lyon or Milwaukee brace. Our results indicate that the use of brace as prescribed may alter the natural history of AIS and JIS. In particular, patients with complete compliance o that removed brace for 1 month of a year (incomplete A) show more favourable outcomes than those that removed brace during school hours (incomplete C) or worn brace overnight only (incomplete D). The type of brace influences the compliance, such that adherence to treatment is higher with PASB than Lyon or Milwaukee brace. Interestingly, AIS patients show a better compliance to bracing than those with JIS. Finally, PASB provides better correction both in adolescent and juvenile curves.

Recent studies have assessed compliance to bracing by either questionnaires [15-18] or a sensor attached to the brace. Notably, compliance measured by sensors was lower compared with that reported by patients [13,22]. In the present investigation, sensors could not be used as they were not available at the time of study beginning. However, the evaluation of compliance was performed by a single surgeon and correlated with clinical signs and information obtained by the patient parents.

Overall, a complete compliance was recorded in 61% of the study sample (51% for juvenile and 64% for adolescent), which is in agreement with the adherence rate reported in the literature (33-97%, mean: 69%).[10,19-25,30,31] Donzelli et al. evaluated the compliance in 68 patients for about five months with an electronic monitor and recorded 91.7% of full compliance. Patients with full-time brace prescription showed a higher adherence than those with part-time bracing. Interestingly, higher compliance rates were observed in patients fulfilling the SOSORT criteria [13].

Brox et al. evaluated compliance and efficacy of bracing without electronic monitor in 495 patients an 389 (79%) patients were registered as compliant, 106 as non-compliant, the progression of curve at long-term were 24% in compliant and 65% in non-compliant, the surgical rate was 3.5% versus 24% [32]. Rahman et al. showed similar results using an electronic monitor to evaluate time bracing in 34 patients and reported curve progression in 11% with high compliance and 56% with low compliance [21].

It is noteworthy that, different from most studies, in the present investigation compliance was monitored for the whole duration of treatment. A more in-depth analysis of our data shows the compliance is usually higher at the beginning of treatment and that the months of non compliance were often in the summer. The greater correction was obtained during the first six months in all the study sample, after which patients with complete compliance continue to improve, while those categorized as incomplete B (brace removed for 2 months of a year) and D show a negative trend, which however did not affect the final outcome. This pattern can be explained by the motivation of the patient is initially higher and gradually decreases during the course of treatment. These considerations may also explain the satisfactory results and the low rate of surgery (2.4% in group that removed brace during school hours (incomplete C), 5.1% in group that worn brace overnight only (incomplete D) and 0% in the other groups).

The point of strengths of this study are the large study sample, the long term follow-up, the radiological evaluation, the different type of braces studied and the management of patients according to the SOSORT guidelines.

## Conclusion

Use the brace as prescribed may alter the natural history of AIS and JIS and curve progression and referral to surgery are lower in patients with high brace compliance. Bracing discontinuation up to 1 month a year does not impact on the treatment outcome. Conversely, wearing the brace only overnight and bracing discontinuation up to 2 months a year is associated with a high rate of curve progression.

## Abbreviations

JIS: Juvenile idiopathic scoliosis; AIS: Adolescent idiopathic scoliosis; SRS: Scoliosis research society; SOSORT: Society on scoliosis orthopedic and rehabilitation treatment; PASB: Progressive action short brace; CM: Curve magnitude; LL: Latero-lateral; AP: Antero-posterior; TA: Torsion of the apical vertebra.

## Competing interests

The authors declare that they have no competing interests.

## Authors’ contributions

AGA participated in the conception, design and coordination, and to acquisition of data, analysis and interpretation of data and drafted the manuscript. MG, FF and VG helped to draft the manuscript. AP participated in the design of the study and performed the statistical analysis. EM participated in the design of the study and helped to draft the manuscript. All authors read and approved the final manuscript.
